# Stabilizing the Li_1.3_Al_0.3_Ti_1.7_(PO_4_)_3_|Li Interface for High Efficiency and Long Lifespan Quasi‐Solid‐State Lithium Metal Batteries

**DOI:** 10.1002/cssc.202200038

**Published:** 2022-04-22

**Authors:** Zhen Chen, Dominik Stepien, Fanglin Wu, Maider Zarrabeitia, Hai‐Peng Liang, Jae‐Kwang Kim, Guk‐Tae Kim, Stefano Passerini

**Affiliations:** ^1^ Helmholtz Institute Ulm (HIU) Helmholtzstrasse 11 89081 Ulm Germany; ^2^ Karlsruhe Institute of Technology (KIT) P.O. Box 3640 76021 Karlsruhe Germany; ^3^ Department of Energy Convergence Engineering Cheongju University Chungbuk 28503 Cheongju Republic of Korea

**Keywords:** batteries, electrolytes, interfacial stability, ionic liquids, lithium

## Abstract

To tackle the poor chemical/electrochemical stability of Li_1+*x*_Al_*x*_Ti_2‐*x*_(PO_4_)_3_ (LATP) against Li and poor electrode|electrolyte interfacial contact, a thin poly[2,3‐bis(2,2,6,6‐tetramethylpiperidine‐*N*‐oxycarbonyl)norbornene] (PTNB) protection layer is applied with a small amount of ionic liquid electrolyte (ILE). This enables study of the impact of ILEs with modulated composition, such as 0.3 lithium bis(fluoromethanesulfonyl)imide (LiFSI)‐0.7 *N*‐butyl‐*N*‐methylpyrrolidinium bis(fluoromethanesulfonyl)imide (Pyr_14_FSI) and 0.3 LiFSI‐0.35 Pyr_14_FSI‐0.35 *N*‐butyl‐*N*‐methylpyrrolidinium bis(trifluoromethanesulfonyl)imide (Pyr_14_TFSI), on the interfacial stability of PTNB@Li||PTNB@Li and PTNB@Li||LiNi_0.8_Co_0.1_Mn_0.1_O_2_ cells. The addition of Pyr_14_TFSI leads to better thermal and electrochemical stability. Furthermore, Pyr_14_TFSI facilitates the formation of a more stable Li|hybrid electrolyte interface, as verified by the absence of lithium “pitting corrosion islands” and fibrous dendrites, leading to a substantially extended lithium stripping‐plating cycling lifetime (>900 h). Even after 500 cycles (0.5C), PTNB@Li||LiNi_0.8_Co_0.1_Mn_0.1_O_2_ cells achieve an impressive capacity retention of 89.1 % and an average Coulombic efficiency of 98.6 %. These findings reveal a feasible strategy to enhance the interfacial stability between Li and LATP by selectively mixing different ionic liquids.

## Graphical abstract

**Incorporating a small amount** of ionic liquid electrolyte (0.3LiFSI‐0.35Pyr_14_FSI‐0.35Pyr_14_TFSI) within a hybrid electrolyte that also contains Li_1.3_Al_0.3_Ti_1.7_(PO_4_)_3_ (LATP) and the polymer PVDF‐TrFE yields drastically enhances interfacial stability against lithium metal, prolonging the lithium stripping‐plating lifetime by a factor of 3.7 and giving superior cycling stability over 500 cycles (0.5C) in full cells with LiNi_0.8_Co_0.1_Mn_0.1_O_2_ (NCM_811_) as the cathode material.

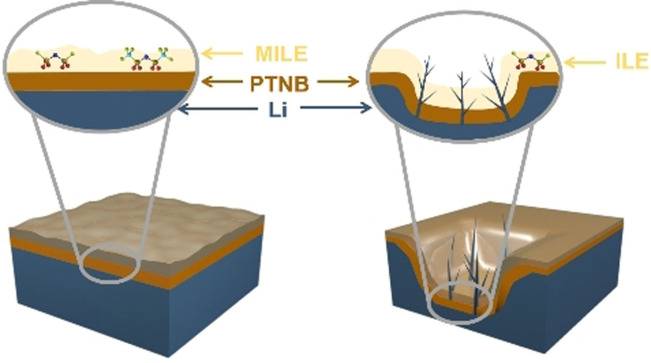

## Introduction

The development of next‐generation high‐performance battery systems hinges on the advancement of science and technology that enables the use of lithium metal as the anode.^[1]^ Despite its appealing features‐high theoretical specific capacity (3860 mAh g^−1^) and low electrochemical potential (−3.04 V vs. standard hydrogen electrode)^[2]^ ‐the severe safety issues and rather poor reversibility due to the inhomogeneous lithium deposition as well as consumption of conventional carbonate‐based organic liquid electrolytes (LEs) at the electrode|electrolyte interface have impeded the development and commercialization of Li metal batteries.^[3]^

One of the most promising approaches to overcome these challenges is to utilize nonflammable solid‐state electrolytes featuring with intrinsic high safety characteristics. Among the options, the NASICON‐type Li_1+*x*_Al_*x*_Ti_2‐*x*_(PO_4_)_3_ (LATP) solid‐state electrolyte, which endows with wide electrochemical stability window, moderately high ionic conductivity and good resistance against air, appears as a promising candidate.^[4]^ Additionally, compared to other promising candidates, such as garnet Li_7_La_3_Zr_2_O_12_ (LLZO) and sulfide‐based Li_10_GeP_2_S_12_ (LGPS), the cheaper LATP renders it more competitive toward mass production and commercial application.^[5]^ However, LATP suffers from chemical/electrochemical instability against lithium metal on account of the formed mixed ionic‐electronic conducting interphase.^[6]^ In view of this issue, numerous efforts have been taken to avoid the direct contact between LATP and Li by introducing an intermediate protection layer made of, for example, germanium,^[7]^ boron nitride,^[8]^ ZnO,^[9]^ nanocomposites (LiF, MgF_2_ and B_2_O_3_),^[10]^ Li_3_PO_4_,^[11]^ or cross‐linked poly(ethylene glycol) methyl ether acrylate (CPMEA).^[12]^

Another remaining challenge of LATP lies in the poor interfacial contact with the electrodes resulting in high overall charge transfer impedances. The employment of solid‐liquid hybrid electrolytes can substantially reduce the electrode|electrolyte contact impedance resulting from the improved interface wetting.^[13]^ However, the carbonate‐based LE decomposition leads to extensive battery performance decay due to the formation of a resistive solid‐liquid electrolyte interphase, especially in the presence of trace water.^[6a]^ Ionic liquid electrolytes (ILEs), on the other hand, endow with high compatibility against lithium metal.^[14]^ Furthermore, ILE exhibits advantages of negligible vapor pressure and very low flammability, high chemical and thermal stability, and in some cases, hydrophobicity.^[15]^ According to Pervez et al.,^[16]^ the application of 0.2LiTFSI‐0.8Pyr_14_FSI ILE thin interlayers on the surface of lithium lanthanum zirconate (LLZO) resulted to substantially lowered interfacial impedances (and thus overpotentials) during lithium stripping‐plating tests of symmetric Li||Li cells and charge‐discharge tests of Li||LiFePO_4_ cells. Although the introduction of the ILE interlayer may raise the overall battery cost, it certainly enables improved performance even avoiding the need of applying external compression to the cells. Furthermore, reduced price is expected when large scale production of ILE is realized for commercial batteries.^[17]^ Strategies such as extending equipment life and economies of scale can mitigate the cost issue.^[18]^

Recently,^[19]^ we revealed that a poly[2,3‐bis(2,2,6,6‐tetramethylpiperidine‐*N*‐oxycarbonyl)‐norbornene] (PTNB) interlayer impregnated with the 0.4LiFSI‐0.6Pyr_14_FSI ILE strongly enhances the cycle ability of the Li metal electrode against LATP‐based hybrid electrolyte. However, the impact of using multi‐anion ILEs, which have been found to have beneficial synergic effects, remains to be studied.^[20]^

It is known that Pyr_14_TFSI exhibits higher stability towards oxidation with respect to Pyr_14_FSI, further allowing for an increased tolerance with the lithium metal electrode by inhibiting the electrolyte degradation during cycling.^[21]^ A recent work done by Wu et al. revealed a synergistic interplay of FSI and TFSI dual anions which enables highly favorable interfacial passivation layers on the surface of both Li metal and Ni‐rich NCM electrodes, contributing to an outstanding capacity retention of 88 % over 1000 cycles.^[22]^

Thus, we hereby compare the performance of hybrid electrolytes employing different ILEs, in particular, the single anion 0.3LiFSI‐0.7Pyr_14_FSI (ILE) and the mixed anion 0.3LiFSI‐0.35Pyr_14_FSI‐0.35Pyr_14_TFSI (MILE). The resulting hybrid electrolytes are denoted as H‐ILE and H‐MILE, accordingly. As expected, the H‐MILE is found to provide a larger electrochemical stability window (i. e., higher oxidation and lower reduction potentials) benefiting from the presence of TFSI anions.^[23]^ Moreover, the decomposition of the two anions contributes to the formation of a thinner, but LiF‐rich interphase. This regulates the homogeneous Li^+^ deposition and stripping and thus mitigates the irreversible interfacial reactions between LATP and Li extending the lithium stripping‐plating cycling lifetime. Due to slightly reduced ionic conductivity and higher viscosity, the rate capacity is unfortunately sacrificed as revealed in cells comprising PTNB‐coated lithium (PTNB@Li), LiNi_0.8_Co_0.1_Mn_0.1_O_2_ (NCM_811_) and H‐MILE hybrid electrolyte, (PTNB@Li|H‐MILE|NCM_811_). However, the average Coulombic efficiency (CE) upon cycling at 0.5C is greatly ameliorated, achieving 99.3 % after 200 cycles and 98.6 % after 500 cycles. The remarkable cycling stability is associated to the stable interfacial impedance upon cycling. Taken together, the results reported herein demonstrate that enhancing the interfacial stability between Li and LATP by mixing different ILs is a feasible strategy. Such a method is expected to be also applicable to other solid‐state electrolytes suffering from poor stability against lithium metal.

## Results and Discussion

**Figure 1:**
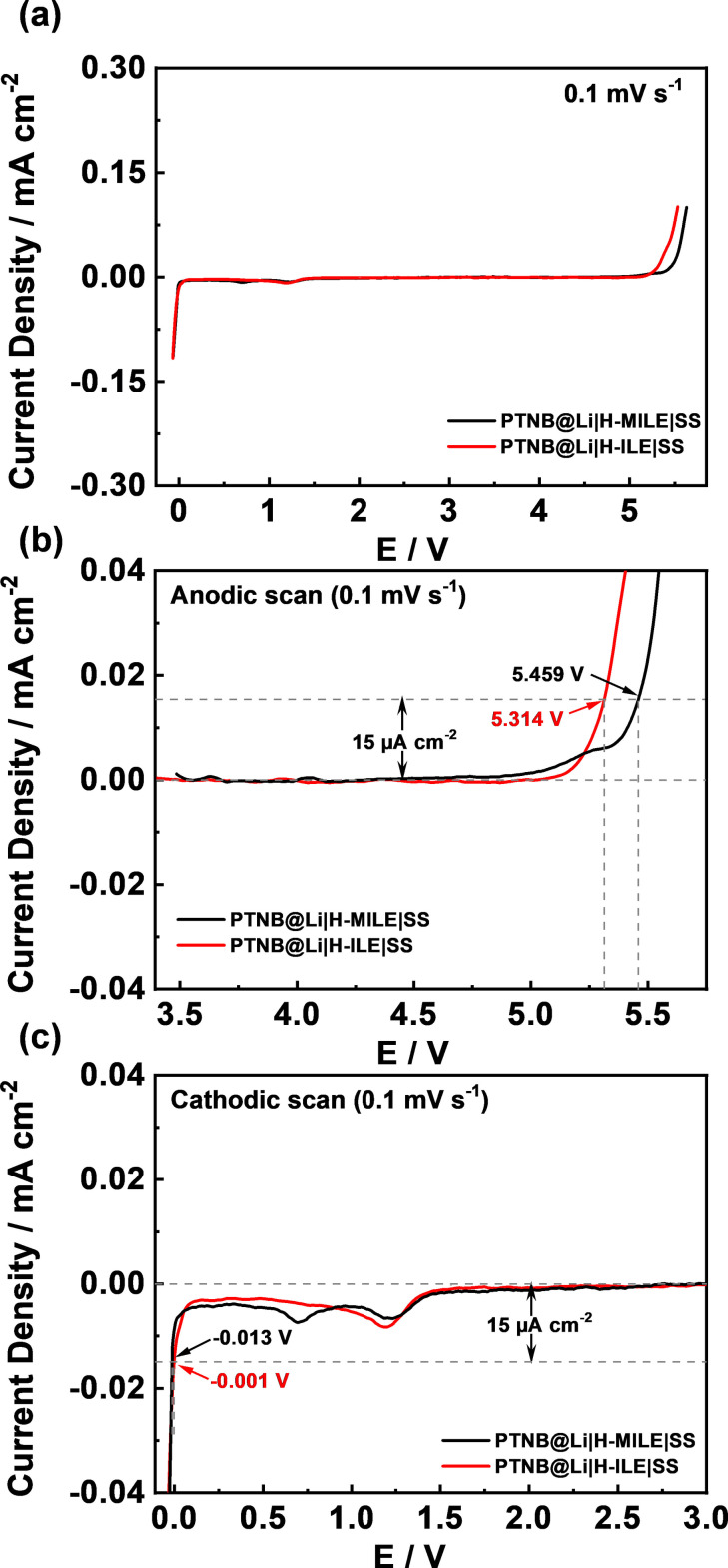
(a) Overall electrochemical stability window and magnifications of the (b) anodic sweep and (c) cathodic sweep of H‐ILE and H‐MILE hybrid electrolytes (*T*=20 °C).

Figure 1a compares the electrochemical stability window (ESW) of the H‐ILE and H‐MILE hybrid electrolytes determined by anodic (Figure 1b) and cathodic (Figure 1c) linear sweep voltammetries. Setting the threshold for the current density flow at 15 μA cm^−2^, the anodic stability of H‐MILE is revealed to be as high as 5.459 V, which is 0.1 V higher than that of the H‐ILE. This is in a good agreement with previous reports showing that the TFSI anion is more stable toward oxidation than FSI anion.[^20c^, ^23^, ^24^] The reduction peak observed at ∼1.2 V in the cathodic scan of H‐ILE is assigned to the formation of the solid electrolyte interphase (SEI) as well as the contribution from impurity present in LiFSI.[^19^, ^20c^, ^23^] In the H‐MILE hybrid electrolyte, this peak shows a mild shift towards higher potential whereas its intensity reduces presumably owing to a lower amount of FSI anions. Additionally, a reduction peak appears at ∼0.7 V, which must be associated with Pyr_14_TFSI. Both H‐MILE and H‐ILE enable lithium plating with a rather low overvoltage. In fact, a current density flow of −15 μA cm^−2^ is attained at −0.013 V and −0.001 V, respectively. The better stability towards reduction is also in a good agreement with literature.[^22^, ^25^] The better oxidation and reduction stability of H‐MILE implies that combining Pyr_14_FSI, Pyr_14_TFSI and LiFSI into a ternary ionic liquid electrolyte may lead to better electrode|electrolyte interfacial compatibility and a wider operating voltage range.

**Figure 2:**
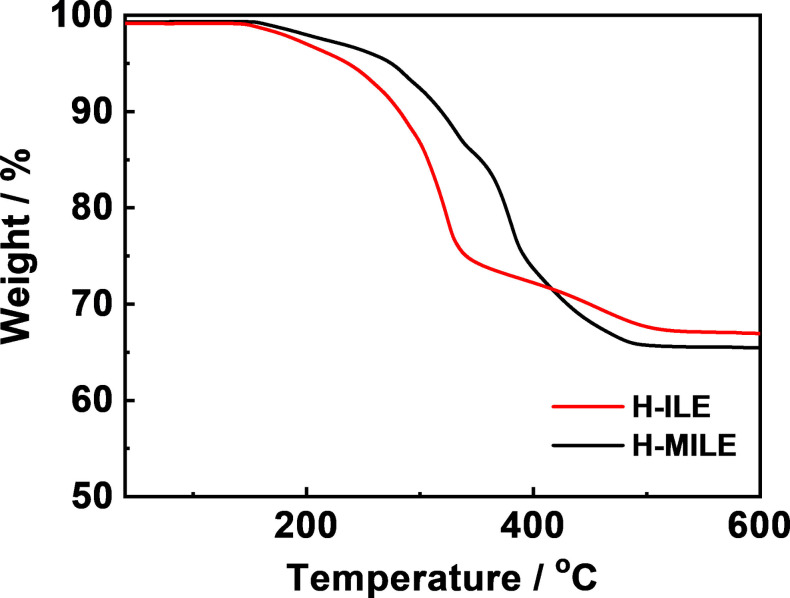
TGA profiles of H‐ILE (red curve) and H‐MILE (black curve) in artificial, oxygen rich air (total flow: 25 mL min^−1^; N_2_: 15 mL min^−1^; O_2_: 10 mL min^−1^).

The thermal stability of H‐ILE and H‐MILE (Figure 2) is determined by thermogravimetric analysis (TGA). By defining the starting decomposition temperature (*T*_start_) as the temperature at which 1 wt % of the sample weight is lost, the *T*_start_ is revealed to be 175 °C and 189 °C for H‐ILE and H‐MILE, respectively. Above 335 °C, an additional slope is evidenced for H‐MILE, attributed to the decomposition of the TFSI anion. Besides of a higher *T*_start_, the decomposition of H‐MILE occurs at higher temperatures highlighting its improved thermal stability. The higher weight loss of H‐MILE compared to H‐ILE (ca. 1.5 wt %) is attributed to a higher density of MILE (1.442 g cm^−3^ vs. 1.409 g cm^−3^) resulting in its larger weight fraction in the hybrid electrolyte (given that the porosity of the LATP/PVDF‐TrFE film is the same for both hybrid electrolytes).

**Figure 3:**
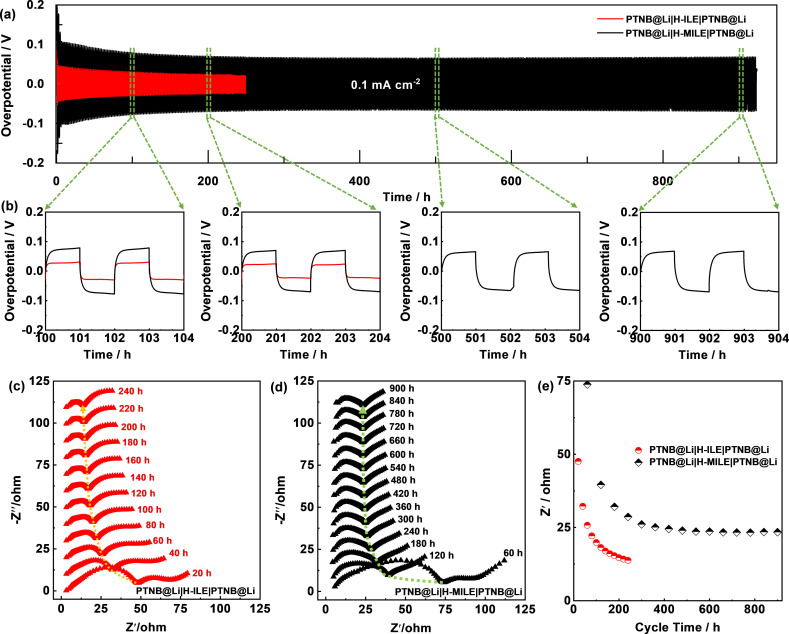
(a) Lithium stripping‐plating tests of PTNB@Li|H‐ILE|PTNB@Li and PTNB@Li|H‐MILE|PTNB@Li cells. (b) Magnification of selected lithium stripping‐plating profiles of PTNB@Li|H‐ILE|PTNB@Li and PTNB@Li|H‐MILE|PTNB@Li cells along the test. (c,d) Selected Nyquist plots: (c) PTNB@Li|H‐ILE|PTNB@Li and (d) PTNB@Li|H‐MILE|PTNB@Li cells upon lithium stripping‐plating test (*T*=20 °C). (e) *Z*′ values versus cycle number collected at 69.3 Hz from Figure 3c and Figure 3d.

Lithium stripping‐plating tests (Figure 3) were performed in symmetric PTNB@Li|H‐MILE|PTNB@Li or PTNB@Li|H‐ILE|PTNB@Li cells to evaluate the interfacial stability of the hybrid electrolytes with lithium metal. The overpotential of the PTNB@Li|H‐ILE|PTNB@Li cell (Figure 3a) decreases upon cycling from 45 mV (2^nd^ cycle) to 20 mV (125^th^ cycle). However, the cell failed due to internal short circuit right after, achieving a cycling lifetime of 250 h. The large initial overpotential is induced by the formation of a rather resistive interphase resulting from the native passivation film on the Li foil and the SEI spontaneously formed through the interaction between Li and ILE.^[26]^ Afterwards, the partial breakdown of the native as well as the newly formed SEI lead to sharp overpotential reduction during initial few cycles.^[26]^ The continuous decrease of overpotential upon consecutive cycling, is primarily attributed to increased surface area as a result of the formation of fibrous dendrites and corrosion pits (see below). The PTNB@Li|H‐MILE|PTNB@Li cell, on the other hand, exhibits a substantially higher overpotential (133 mV at the 2^nd^ cycle). After 290 h, the overpotential gradually reduces to 64 mV. The overall higher overpotential in the PTNB@Li|H‐MILE|PTNB@Li cell can be explained by the lower ionic conductivity of H‐MILE, 0.76 mS cm^−1^, with respect to H‐ILE, 1.48 mS cm^−1^ (see the Supporting Information; Figure S1). However, no apparent overpotential growing is observed afterwards, achieving a highly stable cycling performance up to 924 h before soft short circuits were observed (Figure 3b). This indicates that the substitution of H‐ILE with H‐MILE enables a substantially extended lithium stripping‐plating lifetime. The significantly prolonged cycling lifetime implies that the detrimental side reactions at the interface between PTNB@Li and H‐MILE hybrid electrolyte are effectively suppressed.

To exclude the impact from the PTNB coating layer, we also performed the same lithium stripping‐plating test for Li|H‐MILE|Li and Li|H‐ILE|Li cells (Figure S2). Overall, the overpotential evolution shows a very similar trend to that seen in Figure 3a. Nonetheless, generally smaller overpotentials are observed when avoiding the PTNB coating layer. This is consistent with our previous finding that the introduction of PTNB allows for extended cycling lifetime, but meanwhile increased overpotential.^[19]^ The cycling lifetime is estimated to be 396 h and 108 h for Li|H‐MILE|Li and Li|H‐ILE|Li cells respectively. Given the above results, the superiority of employing H‐MILE over H‐ILE is consolidated in both scenarios with and without the application of the PTNB coating layer.

To understand the reason for the enhanced cycling stability of the cells using H‐MILE, electrochemical impedance spectroscopy (EIS) measurements were performed on PTNB@Li|H‐ILE|PTNB@Li and PTNB@Li|H‐MILE|PTNB@Li cells upon cycling. Figure 3c and Figure 3d show the selected Nyquist plots of the PTNB@Li|H‐ILE|PTNB@Li cell (one every 10 cycles) and the PTNB@Li|H‐MILE|PTNB@Li cell (one every 30 cycles), respectively. The overall impedance of the former cell is smaller than that of the latter one, in accordance with the smaller overpotential observed in Figure 3a. The depressed semicircle from high‐to‐medium frequency is assigned to the impedance of the PTNB@Li|H‐ILE or PTNB@Li|H‐MILE interface. This feature is seen to gradually shrink for the PTNB@Li|H‐ILE|PTNB@Li cell, probably due to the roughening of the electrode surface upon lithium plating and stripping, initially resulting in the increase of the electrochemically active area, but later to the occurrence of soft circuit. By contrast, the interfacial impedance of PTNB@Li|H‐MILE demonstrates a steady drop till 240 h and remains nearly constant afterwards. The evolution of *Z*′ values (collected at 69.3 Hz) upon cycling is shown in Figure 3e. In both cases, the evolution of interfacial impedance is perfectly in line with the overpotential evolution trend (Figure 3a,b). The EIS results reveal a more stable interface formed between PTNB@Li and H‐MILE hybrid electrolyte, which inhibits the degradation of the Li electrode interface and prevents the formation and growth of dendrites.

**Figure 4:**
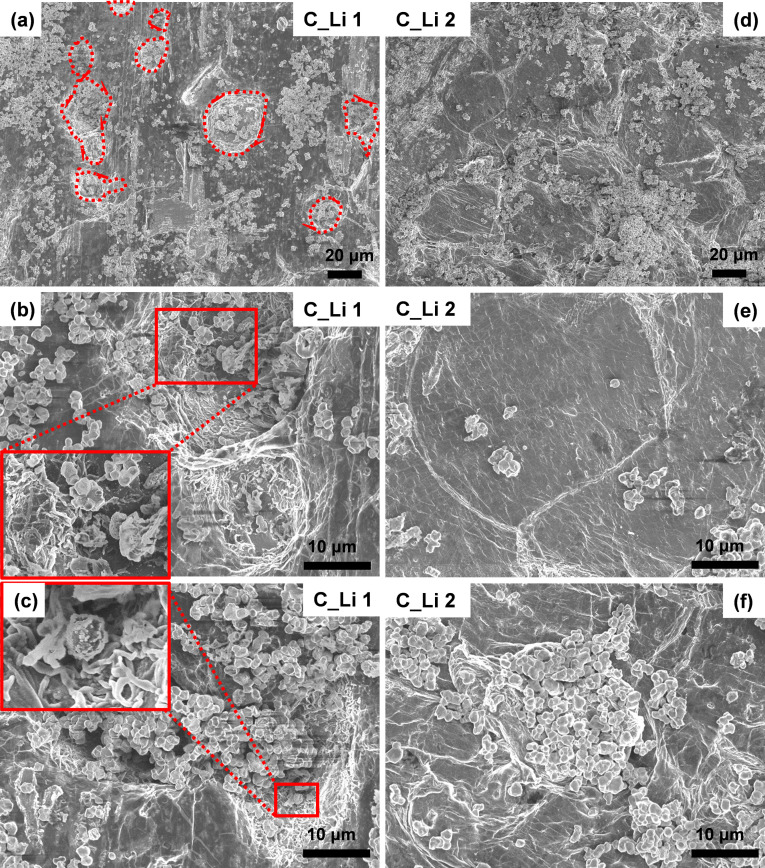
Ex situ surface morphology analysis of cycled PTNB@Li recovered from (a–c) PTNB@Li|H‐ILE|PTNB@Li (denoted as C_Li **1**) and (d–f) PTNB@Li|H‐MILE|PTNB@Li (denoted as C_Li **2**) cells (*T*=20 °C).

The ex situ surface morphology of cycled lithium electrodes was subsequently observed by scanning electron microscope (SEM) investigation (Figure 4 and Figure S3). The lithium metal electrodes recovered from the cycled PTNB@Li|H‐ILE|PTNB@Li and PTNB@Li|H‐MILE|PTNB@Li cells (see Figure 3) are hereby denoted as C_Li **1** and C_Li **2**, respectively. Notably, at low magnification (Figure S3), residual LATP particles are observed on the surface of both cycled lithium electrodes. This could be due to partial breakdown of the native passivation layer, PTNB protection interlayer, and SEI formed upon cycling as a result of huge volume changes of lithium metal. The surface of C_Li **1** shows many “pitting corrosion islands” (red dashed circles in Figure 4a and Figure S3a). Taking a closer look at these “islands” (Figure 4b, c), aggregated LATP particles are found to penetrate into the lithium foil, while rather large and fibrous lithium dendrites are observed on the walls of pits. This underlines that strong parasitic reactions occurred between LATP and lithium metal. The inferior result obtained here comparing to our previous study which used 0.4LiFSI‐0.6Pyr_14_FSI ILE‐based hybrid electrolyte^[19]^ implies that the high LiFSI concentration might play a positive impact on strengthening the interfacial stability of LATP and Li. Consequently, the formation of dendrites and pits enlarges the surface area reducing gradually the overpotential (Figure 3a) and eventually leading to the internal short circuit. In a sharp contrast, the majority surface of C_Li **2** sample (Figure 4d, e) remains rather clean and smooth. Albeit aggregated LATP particles are also observed, they locate on the surface region of lithium metal instead of penetrating into the bulk. Taking a closer look at a representative region where aggregated LATP particles are located (Figure 4f), the lithium surface appears to be rough and uneven. Nevertheless, no fibrous lithium dendrites are observed beneath these agglomerates of LATP particles. The C_Li **2** sample was recovered from the cell undergoing a much longer lithium stripping‐plating cycling (i. e., ca. 924 h vs. ca. 250 h for C_Li **1**). Even under such circumstances, C_Li **2** exhibits substantially better‐preserved surface morphology than C_Li **1**. Therefore, the incorporation of MILE favors the establishment of a stabilized interface between Li metal and the hybrid electrolyte, preventing the formation and growth of lithium dendrites.

**Figure 5:**
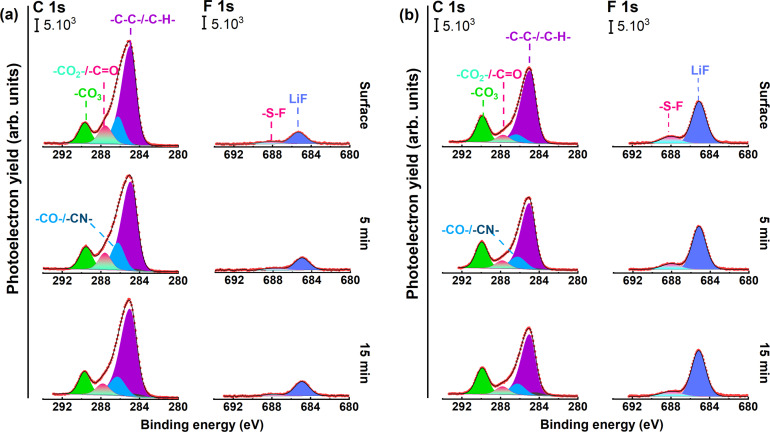
C1s and F1s photoelectron spectra at different depths (surface and after 5 and 15 min Ar^+^ sputtering) of cycled PTNB@Li recovered from (a) PTNB@Li|H‐ILE|PTNB@Li and (b) PTNB@Li|H‐MILE|PTNB@Li cells.

In the next step, X‐ray photoelectron spectroscopy (XPS) was conducted to analyze the interphase formed on the surface of PTNB@Li in H‐ILE and H‐MILE (Figure 5: C1s and F1s regions; Figure S4: N1s and S2p regions). The interphase formed on lithium with both the electrolytes is similar in terms of chemical composition also at different depths. Specifically, the interphase species are hydrocarbons (−C−C−/−C−H−) and CN‐containing species from IL decomposition demonstrated by the C1s,^[27]^ FSI (or FSI and TFSI) and their decomposition reaction products such as reduced −SO_2_N(−)−SO_2_−, Li_3_N, −SO_*x*_−, Li_2_S, and LiF, as indicated by the F1s, N1s and S2p XP spectra.[^19^, ^28^] Moreover, the surfaces of both PTNB@Li contain carbon‐oxygen species, which corresponds to PTNB polymer and suggest that the formed SEI is inhomogeneous and/or rather thin. In addition, the depth profiling study suggests that both formed interphases are stable in the range of 12 nm in depth. It is worth mentioning that the interphases formed in both samples are thicker than 12 nm because the decomposition products are still observed after 15 min of Ar^+^ sputtering. Nevertheless, the better electrochemical performance of H‐MILE might be related with the formed fluorine‐rich interphases, for example, LiF and reduced species of FSI/TFSI anions such as ‐SO_2_N(−)−SO_2_−. In order to elucidate that the higher concentration of fluorine species is not due to the longer cycling time of PTNB@Li|H‐MILE|PTNB@Li cell, we performed the XPS measurements on the Li electrodes extracted from two PTNB@Li|H‐ILE|PTNB@Li and PTNB@Li|H‐MILE|PTNB@Li cells, which were cycled for the same time (150 h). PTNB@Li cycled with H‐MILE showed a higher LiF concentration than that cycled with H‐ILE (Figure S5). It is known that LiF is formed at the initial stages of electrolyte decomposition and subsequent bonding with Li surface.^[29]^ Therefore, LiF is usually observed at higher depth, close to the Li metal surface.[^19^, ^29b^] On the other hand, the C1s photoelectron spectra show that the Pyr^14+^ decomposed more intensively in the H‐ILE, resulting in higher concentration of hydrocarbons and CN‐containing species. Therefore, the possible explanation for the highest LiF concentration is that the interphase of PTNB@Li tested in H‐MILE system is thinner. LiF is known as a good electrical insulator, effectively blocking the interfacial side reactions, meanwhile enabling homogeneous Li^+^ flux at the interface.^[30]^ Therefore, the thinner, but LiF‐rich interphase benefits for suppressing lithium dendrite growth and preventing pitting corrosion, enabling a better PTNB@Li|hybrid electrolyte interface and interphase.^[30b]^

**Figure 6:**
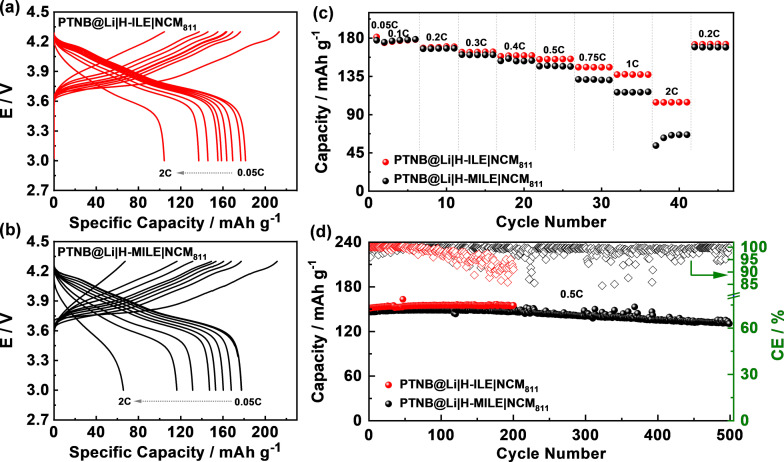
Selected (dis)charge voltage profiles at different C‐rates (0.05C–2C) of (a) PTNB@Li|H‐ILE|NCM_811_ and (b) PTNB@Li|H‐MILE|NCM_811_ cells. Comparative (c) rate capability and (d) cycling performance of PTNB@Li|H‐ILE|NCM_811_ and PTNB@Li|H‐MILE|NCM_811_ cells (*T*=20 °C).

The above characterizations and analyses clearly demonstrate that H‐MILE exhibits higher compatibility with lithium metal. Thus, the electrochemical performance of full cells comprising PTNB@Li as the negative electrode, NCM_811_ as the positive electrode and H‐MILE (or H‐ILE for the sake of comparison) as the electrolyte, was investigated. Figure 6a and 6b display selected (dis)charge voltage profiles of PTNB@Li|H‐ILE|NCM_811_ and PTNB@Li|H‐MILE|NCM_811_ cells at various C‐rates. Initially, the PTNB@Li|H‐ILE|NCM_811_ cell delivers a slightly higher capacity than the PTNB@Li|H‐MILE|NCM_811_ cell (ca. 181 vs. 177 mAh g^−1^). Similar capacities are delivered at low current densities (0.1C and 0.2C) by both cells (i. e., 177 vs. 178 mAh g^−1^ at 0.1C and 169 vs. 168 mAh g^−1^ at 0.2C). However, a further increasing of C‐rates from 0.3C to 2C leads to more obvious differences of delivered capacities. Specifically, PTNB@Li|H‐ILE|NCM_811_ cell delivers capacities of 155 (0.5C), 146 (0.75C), 137 (1C) and 104 (2C) mAh g^−1^, higher than those obtained by PTNB@Li|H‐MILE|NCM_811_ cell (147 (0.5C), 131 (0.75 C), 116 (1C), and 66 (2C) mAh g^−1^). Particularly, the sudden capacity drop from 1C to 2C and, afterwards, the gradual capacity increase of the PTNB@Li|H‐MILE|NCM_811_ cell (Figure 6c) reflect a high polarization existing in the cell. This is primarily associated with the lower ionic conductivity of H‐MILE with respect to the H‐ILE counterpart (0.76 vs. 1.48 mS cm^−1^; Figure S1) since a fast ion transfer is vital for the performance at high (dis)charge rates.

The evaluation of long‐term cycling stability at 0.5C is shown in Figure 6d. In line with the rate capability result, the PTNB@Li|H‐MILE|NCM_811_ and PTNB@Li|H‐ILE|NCM_811_ cells deliver an initial capacity of 147 and 151 mAh g^−1^ after the formation cycles. The discharge capacity gradually increases upon cycling and stabilizes afterwards in both cells, achieving capacities of 149 and 155 mAh g^−1^ after 200 cycles. Notably, the PTNB@Li|H‐MILE|NCM_811_ cell shows an apparent superiority over PTNB@Li|H‐ILE|NCM_811_ cell in terms of CE (i. e., 99.3 % vs. 96.9 % after 80 cycles). The high CE of the PTNB@Li|H‐MILE|NCM_811_ cell is comparable to (or even superior to) that of other quasi‐solid‐state electrolytes.^[31]^ The lower CE of PTNB@Li|H‐ILE|NCM_811_ cell is related to the formation of small lithium dendrites generating soft short circuits, which lead to extra charge capacity whereas the discharge capacity remains rather stable. To verify this, ex situ SEM images of PTNB@Li recovered from PTNB@Li|H‐ILE|NCM_811_ and PTNB@Li|H‐MILE|NCM_811_ cells cycled for 200 cycles were taken. The former shows severe lithium corrosion pits (Figure S6a, b). Additionally, long fibrous Li dendrites are seen (Figure S6c, d), which explain the low CE (Figure 6d). On the contrary, the cycled PTNB@Li recovered from PTNB@Li|H‐MILE|NCM_811_ cell displays a rather smooth surface (Figure S7a, b). A few small Li dendrites are also observed (red arrows in Figure S7c, d). However, the Li dendrite formation is effectively mitigated in the PTNB@Li|H‐MILE|NCM_811_ cell, in good consistency with its higher CE. It again verifies that the employment of H‐MILE substantially promotes the cycling compatibility against PTNB@Li. Such a distinct behavior is highly consistent with the results demonstrated in lithium stripping‐plating test (Figure 3a) and ex situ electrode morphology analysis (Figure 4). Considering the sharp drop of CE, the PTNB@Li|H‐ILE|NCM_811_ cell was terminated after 200 cycles. On the other hand, the PTNB@Li|H‐MILE|NCM_811_ cell was kept cycling for additional 300 cycles. Although soft short circuits seldomly occurred, the overall CE over 500 cycles is rather high, achieving an average value of 98.9 %. Even after 500 cycles, the PTNB@Li|H‐MILE|NCM_811_ cell still delivered a capacity of 131 mAh g^−1^, retaining 89.1 % of the initial capacity. Albeit the inferior performance shown at high current densities, the PTNB@Li|H‐MILE|NCM_811_ cell enables much better long‐term cyclability regarding to both capacity and CE, which are critical in practical applications with a limited lithium inventory.

**Figure 7:**
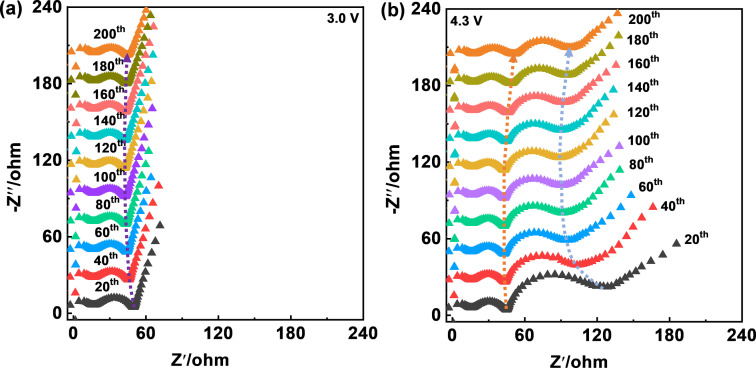
Selected Nyquist plots (every 20 cycles) of a PTNB@Li|H‐MILE|NCM_811_ cell recorded at (a) fully discharged state (3.0 V) and (b) fully charged state (4.3 V) over 200 cycles (*T*=20 °C).

For the further investigation of the stable cycling performance achieved by PTNB@Li|H‐MILE|NCM_811_ cell, EIS measurements were conducted on the cell discharged to 3.0 V (Figure 7a) and charged to 4.3 V (Figure 7b). Figure 7 shows selected Nyquist plots (one every 20 cycles) over 200 cycles.

At the discharged state (3.0 V), the depressed semicircle has two major contributions.^[19]^ One corresponds to the contact impedance between the Al current collector and the NCM_811_ electrode, which is revealed to be independent of the state‐of‐charge, generally remaining constant upon de‐/lithiation unless noticeable electrode volume changes occur. The second contribution corresponds to the interfacial impedance of PTNB@Li|H‐MILE. Thereby, the nearly constant diameter of the depressed semicircle in Figure 7a suggests a remarkably stable interfacial impedance of PTNB@Li|H‐MILE.

At the charged state (4.3 V), the interfacial impedance of PTNB@Li|H‐MILE slightly increases after 80 cycles, which could be due to the thickening of the interphase on the Li surface. Additionally, the newly emerged depressed semicircle in the medium‐to‐low frequency range is ascribed to the NCM_811_|H‐MILE interfacial impedance. In the initial 60 cycles, the NCM_811_|H‐MILE interfacial impedance gradually decreases, primarily due to the slow electrode activation resulting from the viscous MILE. Afterwards, it stays constant for over 200 cycles, reflecting the high stability of NCM_811_|H‐MILE interface.

## Conclusion

The impact of incorporating different ILEs into hybrid electrolytes on the oxidation/reduction stability, thermal stability, and most importantly, the interfacial stability against lithium metal has been investigated. By replacing the binary 0.3LiFSI‐0.7Pyr_14_FSI with the ternary 0.3LiFSI‐0.35Pyr_14_FSI‐0.35Pyr_14_TFSI, the detrimental interfacial side reactions are effectively reduced as evidenced by the elimination of the lithium “pitting corrosion islands”, the fibrous dendritic lithium, as well as the nearly constant interfacial resistance upon cycling. Thus, the stabilized interface contributes to the significantly improved cycling performance in both symmetric cells and full cells. Impressively, the lithium stripping‐plating lifetime is prolonged by a factor of 3.7. The evaluation of PTNB@Li|H‐MILE|NCM_811_ full cells demonstrates a stable cycling for 500 cycles at 0.5C, retaining 89.1 % of initial capacity and achieving an average CE of 98.9 %. Therefore, the strategy of selectively mixing different ionic liquids may provide new insights for the establishment of stable Li|solid electrolyte interface for high‐performance quasi‐solid‐state lithium metal batteries.

## Experimental Section

The preparation of all electrodes and electrolytes, the assembly of cells and the handling of ex situ samples were conducted in a dry room with a dew point always below −60 °C at 20 °C.

### Preparation of hybrid electrolyte

The preparation of dry LATP/poly(vinylidene fluoride‐trifluorethylene (PVDF‐TrFE) and LATP/PVDF‐TrFE/ILE films follows the same method already described in the previous studies.[^19^, ^32^] A phase inversion process was used to prepare the dry LATP/PVDF‐TrFE . In brief (Figure S8), the homogeneously mixed slurry containing LATP, PVDF‐TrFE/NMP and acetone was cast on a glass plate. After the acetone was evaporated, the glass plate together with the coating sheet was immerged into a water bath. Subsequently, a vacuum drying and a hot calendering step were applied to obtain the dry LATP/PVDF‐TrFE film. Afterwards, 100 μL of ILE (0.3LiFSI‐0.7Pyr_14_FSI) or MILE (0.3LiFSI‐0.35Pyr_14_FSI‐0.35Pyr_14_TFSI) was added into the above‐mentioned LATP/PVDF‐TrFE film (1.5 cm×2 cm) with the aid of ambient vacuum. The excess ILE was squeezed to avoid any free‐flowing liquid electrolyte. The as‐prepared hybrid electrodes are labeled as H‐ILE or H‐MILE, accordingly.

### Preparation of electrodes

The PTNB@Li and NCM_811_ electrodes were prepared according to the previous study.^[19]^ In brief, a piece of lithium strip was dipped into a PTNB solution in 1,2‐dimethoxyethane (DME). After 4 min, the DME solvent was immediately removed by applying a vacuum step. To realize large scale PTNB‐coated Li metal, a spray coating method could be easily adapted. The NCM_811_ slurries were prepared by intimately mixing NCM_811_, Super C65 and PVDF in *N*‐methyl‐2‐pyrrolidone (NMP) with a weight ratio of 92 : 4 : 4. A doctor blade technique was adopted to cast the slurries on aluminum foils. Next, the as‐prepared wet electrodes sheets were pre‐dried in an oven (60 °C) to remove NMP and then fully dried under vacuum (100 °C, 12 h). Prior to cell assembly, the porosity of NCM_811_ electrodes was filled with 5 μL of ILE (or MILE) with the aid of a vacuum step (15 min under a pressure of 10^−3^ mbar at 20 °C). The electrodes were wiped carefully afterwards to eliminate any free‐flowing liquid. The average NCM_811_ mass loading density was calculated to be ∼2.5±0.1 mg cm^−2^. All the specific capacity values are calculated based on the mass of NCM_811_.

### Cell assembly

The ESW of both H‐ILE and H‐MILE was determined in two‐electrode Swagelok cells using a polymer‐coated lithium metal electrode (PTNB@Li) and an ion‐blocking stainless steel (SS) electrode (i. e., PTNB@Li|H‐ILE|SS and PTNB@Li|H‐MILE|SS). The ionic conductivity of H‐ILE and H‐MILE was measured in two‐electrode pouch cells by sandwiching a layer of H‐ILE or H‐MILE between two copper foil electrodes. Lithium stripping‐plating tests were conducted in two‐electrode pouch cells composed of two PTNB@Li electrodes separated by a layer of H‐ILE or H‐MILE as the electrolyte. Accordingly, the cells are named as PTNB@Li|H‐ILE|PTNB@Li or PTNB@Li|H‐MILE|PTNB@Li. Full cells, for example, PTNB@Li|H‐ILE|NCM_811_ (or PTNB@Li|H‐MILE|NCM_811_), were assembled in two‐electrode pouch cells comprising NCM_811_ as the positive electrode, PTNB@Li as the negative electrode and H‐ILE (or H‐MILE) as the electrolyte.

### Materials and electrochemical characterization

The density of ILE and MILE was measured by a density meter (Anton Paar DMA 5000M). The thermal stability of H‐ILE and H‐MILE hybrid electrolytes was examined by TGA (Discovery TGA, TA instruments). The samples were heated up to 600 °C at a heating rate of 3 °C min^−1^ in an artificial air atmosphere. The gas flow rate ratio of N_2_ and O_2_ was fixed at 60 : 40.

All the electrochemical performance tests were performed using a battery tester (Maccor series 4000). The current density for lithium stripping‐plating test is fixed at 0.1 mA cm^−2^ and the time for each cycle is 2 h. For the long‐term cycling test, cells were always activated by one cycle at 0.05C and three cycles at 0.1C before subjecting to 200 cycles (PTNB@Li|H‐ILE|NCM_811_) or 500 cycles (PTNB@Li|H‐MILE|NCM_811_) at 0.5C. The rate capability was evaluated by cycling fresh cells at different (dis)charge rates ranging from 0.05C to 2C. The current density at the 1C rate corresponds to 200 mA g^−1^. The voltage range for full cell test is 3.0–4.3 V. The EIS (frequency range: 1 MHz–10 mHz; AC amplitude: 10 mV) upon lithium stripping‐plating in symmetric cells and galvanostatic cycling in full cells were recorded using a multi‐channel potentiostat (VMP Biologic‐Science Instruments). The cell operation temperature was always fixed at 20 °C, controlled by a climatic chamber (Binder GmbH).

To prepare ex situ samples, for example, cycled PTNB@Li recovered from symmetric cells and PTNB@Li|H‐ILE|NCM_811_ (or PTNB@Li|H‐MILE|NCM_811_) cells, the ILE or MILE was first removed by rinsing with dimethyl carbonate (DMC) solvent, and subsequently applying a vacuum drying step at 20 °C to remove residual DMC. For ex situ SEM/XPS measurements, samples were transferred using a home‐designed air‐tight transfer box to avoid any contact with moist air.

The morphology of cycled lithium foils recovered from symmetric and full cells was investigated by SEM (ZEISS EVO MA 10 microscope). The interphase on lithium metal was also examined by XPS analysis using a monochromatic Al_Kα_ (*hν*=1,487 eV) X‐ray source and a Phoibos 150 XPS spectrometer (SPECS‐Surface concept) equipped with a micro‐channel plate and Delay Line Detector (DLD). The scans were acquired in a Fixed Analyzer Transmission mode with an X‐ray source power of 200 W (15 kV), 30 eV pass energy and 0.1 eV energy steps. The depth profiling was performed by a 5 keV Ar^+^ focused ion gun with an ion filter and sputtering rate of 0.8 nm min^−1^. The CasaXPS software was used for the spectra fitting, using a nonlinear Shirley‐type background and 70 % Gaussian and 30 % Lorentzian profile function.

## Conflict of interest

The authors declare no conflict of interest.

1

## Supporting information

As a service to our authors and readers, this journal provides supporting information supplied by the authors. Such materials are peer reviewed and may be re‐organized for online delivery, but are not copy‐edited or typeset. Technical support issues arising from supporting information (other than missing files) should be addressed to the authors.

Supporting Information

## Data Availability

The data that support the findings of this study are available from the corresponding author upon reasonable request.
