# Supplementary material for: Stabilizing the Li1.3Al0.3Ti1.7(PO4)3|Li Interface for High Efficiency and Long Lifespan Quasi‐Solid‐State Lithium Metal Batteries
Source: ChemSusChem. 2022 Apr 22;15(10):e202200038. doi: 10.1002/cssc.202200038 (PMC9325468; doi:10.1002/cssc.202200038)
Supplement: Supplementary file 1 — Supporting Information [file CSSC-15-0-s001.pdf]

ChemSusChem

Supporting Information

Stabilizing the $Li_{1.3}Al_{0.3}Ti_{1.7}(PO_4)_3$|Li Interface for High Efficiency and Long Lifespan Quasi-Solid-State Lithium Metal Batteries

Zhen Chen, Dominik Stepien, Fanglin Wu, Maider Zarrabeitia, Hai-Peng Liang, Jae-Kwang Kim, Guk-Tae Kim,* and Stefano Passerini*© 2022 The Authors. ChemSusChem published by Wiley-VCH GmbH. This is an open access article under the terms of the Creative Commons Attribution License, which permits use, distribution and reproduction in any medium, provided the original work is properly cited.

Author Contributions

Z.C. Conceptualization:Equal; Data curation:Lead; Investigation:Lead; Writing – original draft:Lead
D.S. Investigation:Supporting; Writing – original draft:Supporting
F.W. Investigation:Supporting; Writing – original draft:Supporting
M.Z. Data curation:Supporting; Investigation:Supporting; Writing – original draft:Supporting
H.-P.L. Investigation:Supporting; Writing – original draft:Supporting
J.-K.K. Funding acquisition:Supporting; Resources:Supporting; Writing – review & editing:Supporting
G.-T.K. Conceptualization:Lead; Data curation:Equal; Investigation:Supporting; Writing – original draft:Supporting
S.P. Conceptualization:Supporting; Resources:Lead; Supervision:Lead; Writing – review & editing:Lead

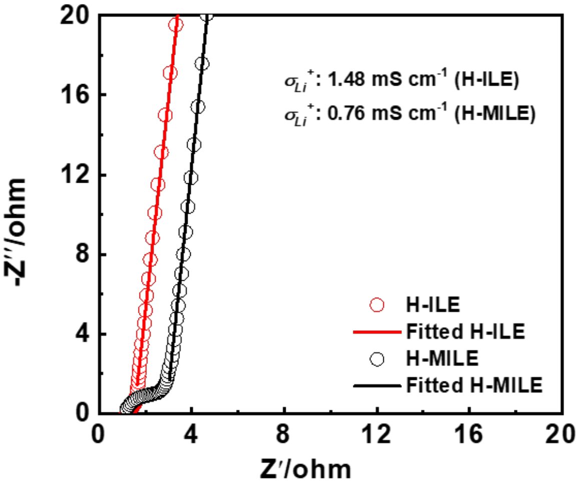

Figure S1. EIS measurements performed on H-MILE and H-ILE hybrid electrolytes (T: 20 °C) between two copper electrodes. The ionic conductivity was determined from the intercept of the low frequency capacitive line with the real axis. Two ion conducting mechanisms exist in the electrolyte as revealed by the high-frequency semicircle (better seen for the H-MILE electrolyte), which does not intercept the real axis in the origin. The two different mechanism are most likely associated with ion mobility in LATP as well as ILE.

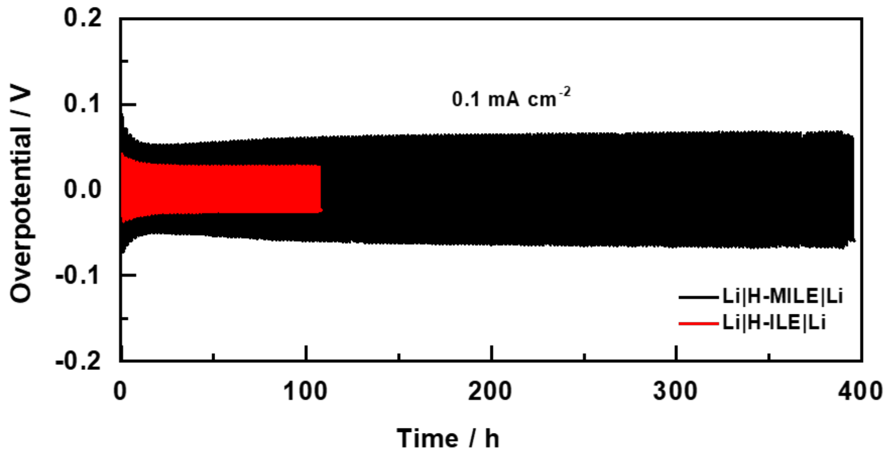

Figure S2. Lithium stripping-plating tests of Li|H-MILE|Li and Li|H-ILE|Li cells (T: 20 °C).

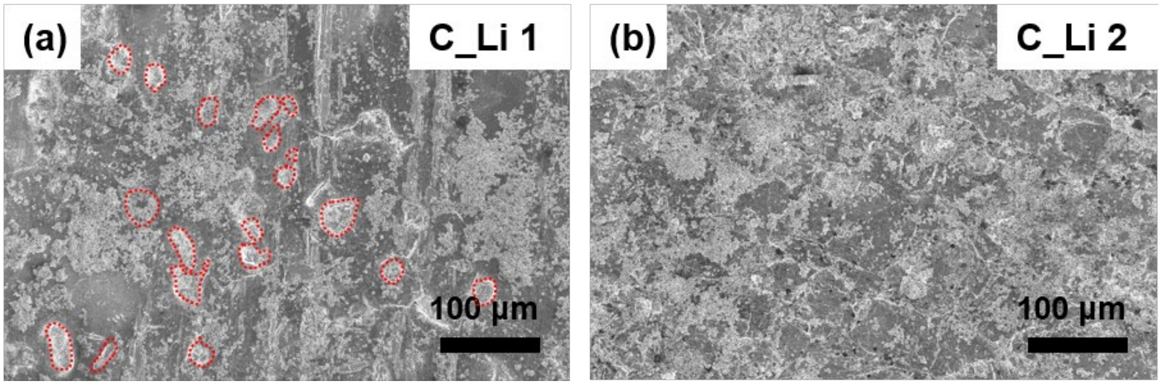

Figure S3. *Ex situ* surface morphology analysis of cycled PTNB@Li recovered from (a) PTNB@Li|H-ILE|PTNB@Li (denoted as C_Li 1) and (b) PTNB@Li|H-MILE|PTNB@Li (denoted as C_Li 2) cells (T: 20 °C).

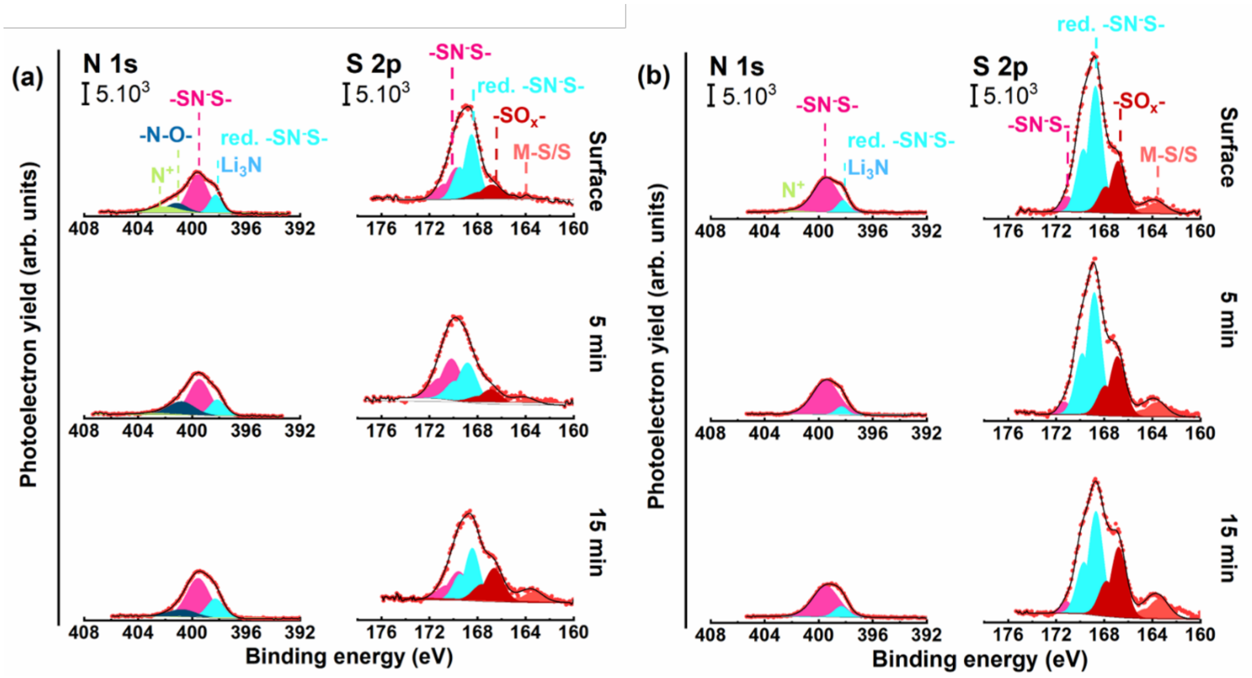

Figure S4. N 1s and S 2p photoelectron spectra at different depths (surface and after 5 and 15 min Ar^+ sputtering) of cycled PTNB@Li recovered from (a) PTNB@Li|H-ILE|PTNB@Li and (b) PTNB@Li|H-MILE|PTNB@Li cells.

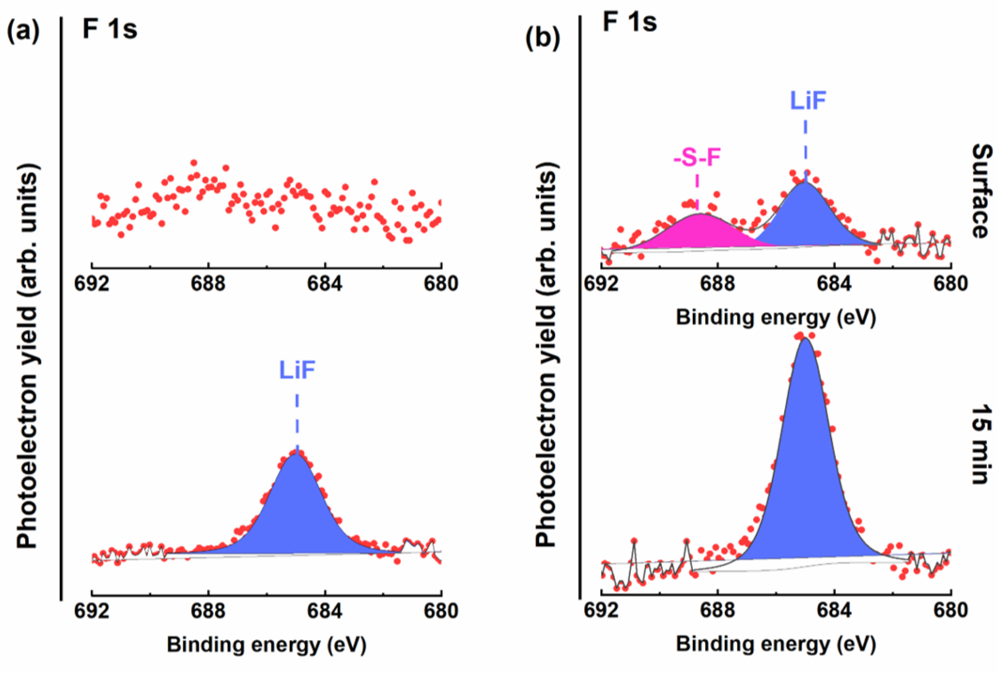

Figure S5. F 1s photoelectron spectra at different depths (surface and after 15 min Ar^{+} sputtering) of cycled PTNB@Li electrodes recovered from PTNB@Li|H-ILE|PTNB@Li and PTNB@Li|H-MILE|PTNB@Li cells respectively after cycling for 150 h.

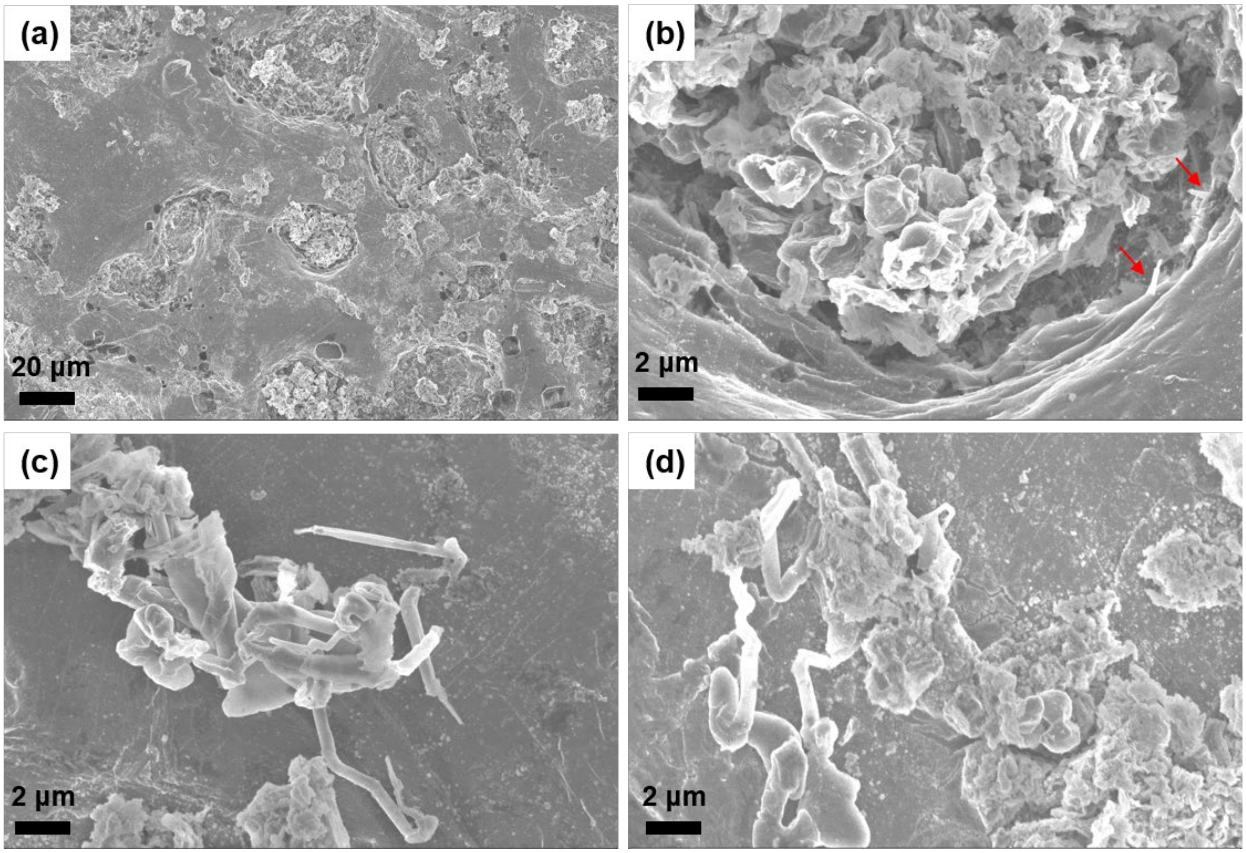

Figure S6. *Ex situ* surface morphology analysis of cycled PTNB@Li recovered from PTNB@Li|H-ILE|NCM_{811} cell (T: 20 °C).

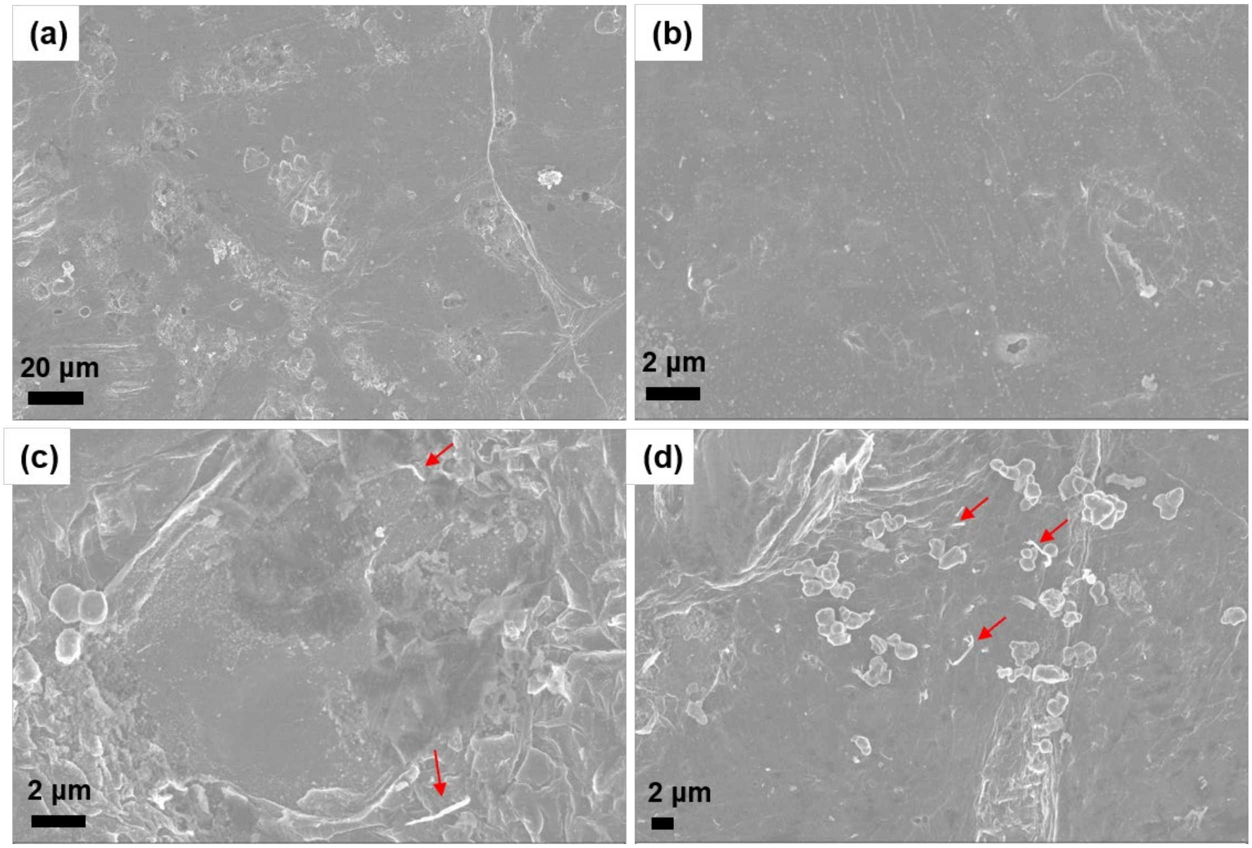

Figure S7. *Ex situ* surface morphology analysis of cycled PTNB@Li recovered from PTNB@Li|H-MILE|NCM_{811} cell (T: 20 °C).

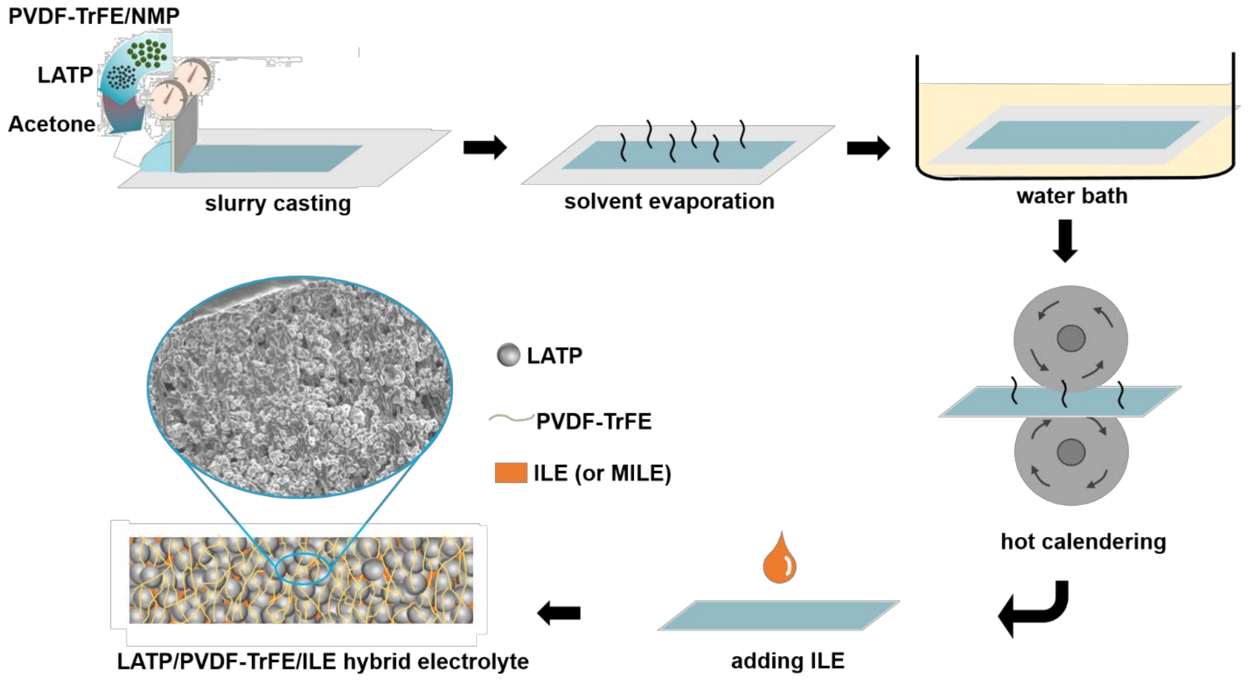

Figure S8. Schematic illustration of the preparation of LATP/PVDF-TrFE/ILE hybrid electrolyte.
